# Protective Effect of Human Leukocyte Antigen (HLA) Allele DRB1*13:02 on Age-Related Brain Gray Matter Volume Reduction in Healthy Women

**DOI:** 10.1016/j.ebiom.2018.02.005

**Published:** 2018-02-08

**Authors:** Lisa M. James, Peka Christova, Scott M. Lewis, Brian E. Engdahl, Angeliki Georgopoulos, Apostolos P. Georgopoulos

**Affiliations:** a Brain Sciences Center, Department of Veterans Affairs Health Care System, Minneapolis, MN 5541, USA; b Department of Neuroscience, University of Minnesota Medical School, Minneapolis, MN 55455, USA; c Department of Psychiatry, University of Minnesota Medical School, Minneapolis, MN 55455, USA; d Center for Cognitive Sciences, University of Minnesota, Minneapolis, MN 55455, USA; e Department of Neurology, University of Minnesota Medical School, Minneapolis, MN 55455, USA; f Department of Psychology, University of Minnesota, Minneapolis, MN 55455, USA; g Department of Medicine, University of Minnesota Medical School, Minneapolis, MN 55455, USA

**Keywords:** Healthy brain aging, Human Leukocyte Antigen, DRB1*13:02, Brain atrophy

## Abstract

**Background:**

Reduction of brain volume (brain atrophy) during healthy brain aging is well documented and dependent on genetic, lifestyle and environmental factors. Here we investigated the possible dependence of brain gray matter volume reduction in the absence of the Human Leukocyte Antigen (HLA) allele DRB1*13:02 which prevents brain atrophy in Gulf War Illness (James et al., 2017).

**Methods:**

Seventy-one cognitively healthy women (32–69 years old) underwent a structural Magnetic Resonance Imaging (sMRI) scan to measure the volumes of total gray matter, cerebrocortical gray matter, and subcortical gray matter. Participants were assigned to two groups, depending on whether they lacked the DRB1*13:02 allele (No DRB1*13:02 group, N = 60) or carried the DRB1*13:02 allele (N = 11). We assessed the change of brain gray matter volume with age in each group by performing a linear regression where the brain volume (adjusted for total intracranial volume) was the dependent variable and age was the independent variable.

**Findings:**

In the No DRB1*13:02 group, the volumes of total gray matter, cerebrocortical gray matter, and subcortical gray matter were reduced highly significantly. In contrast, none of these volumes showed a statistically significant reduction with age in the DRB1*13:02 group.

**Interpretation:**

These findings document the protective effect of DRB1*13:02 on age-dependent reduction of brain gray matter in healthy individuals. Since the role of this allele is to connect to matching epitopes of external antigens for the subsequent production of antibodies and elimination of the offending antigen, we hypothesize that its protective effect may be due to the successful elimination of such antigens to which we are exposed during the lifespan, antigens that otherwise would persist causing gradual brain atrophy. In addition, we consider a possible beneficial role of DRB1*13:02 attributed to its binding to cathepsin S, a known harmful substance in brain aging (Wendt et al., 2008). Of course, other factors covarying with the presence of DRB1*13:02 could be involved.

## Highlights

• The influence of HLA on age-related reduction in brain gray matter was investigated in a sample of cognitively healthy women.
• The DRB1*13:02 allele was found to protect against age-related gray matter atrophy in this sample.
• Other factors, covarying with the presence of DRB1*13:02, could also be involved in this beneficial effect.

Although age-related brain atrophy has been widely reported, there is considerable individual variability, some of which may be accounted for by genetic variation. Here we report on a possible neuroprotective effect of Human Leukocyte Antigen DRB1*13:02 allele in a sample of cognitively healthy women. We found a statistically significant age-related brain volume reduction in a group of women lacking the DRB1*13:02 allele, but no such effect in carriers of this allele. We hypothesize that the protective effect of DRB1*13:02 is related to successful elimination of specific pathogens that would ultimately cause gradual brain atrophy. It is also possible that other factors are involved in this neuroprotection.

## 1 Introduction

Age-related brain changes are widely regarded as inevitable. Reductions in gray matter volume with increasing age have been consistently reported (Raz and Rodrigue, 2006; Lemaitre et al., 2012; Walhovd et al., 2011; Jiang et al. 2014); however, considerable heterogeneity in rates of atrophy have been observed. For instance, prefrontal cortices are more affected than posterior regions (Raz and Rodrigue, 2006) and some subcortical regions (hippocampus, amygdala, cerebellum) evidence more prominent and consistent volumetric reductions than others that seem relatively impervious to age-related changes (e.g., brainstem) (Walhovd et al., 2011). Reports of volumetric white matter reductions with age have been less consistent although compelling evidence suggests a curvilinear association with white matter volume increasing through middle age before decreasing at an accelerated rate (Allen et al., 2005; Raz et al., 2005). Owing to varied methodological differences including sample characteristics and segmentation procedures, results regarding gray matter and white matter volumetric reductions in specific areas have been highly inconsistent across studies. Nonetheless, global atrophy is consistently evident and estimated at 2–5% per decade for normal aging, with rates of loss increasing with age (Enzinger et al., 2005; Fjell et al., 2009; Resnick et al., 2003). This stands in contrast to pathological aging, such as Alzheimer's disease, where rates of 20–30% volume reduction per decade have been reported (Fox et al., 1999). Notably, the rate of atrophy is reduced among the very healthy elderly (Resnick et al., 2003); however, even among cognitively healthy individuals, there is considerable variability in rates of atrophy (Raz et al., 2010).

Numerous possible modifiers of age-related atrophy have been investigated with mixed findings. Among the most consistently investigated and supported individual difference factors that contribute to variability in brain atrophy are health factors such as hypertension, diabetes mellitus, alcohol consumption, smoking, vascular pathology, and stress (Enzinger et al., 2005; Raz and Rodrigue, 2006). Notably, all of these factors have been linked to inflammation which, when chronic, damages the brain and other organs, particularly among genetically vulnerable individuals (Licastro et al., 2005). In terms of genetic influence on age-related brain changes, perhaps the most widely studied contributor of age-related brain changes is apolipoprotein E (apoE) E4, a well-established Alzheimer's disease susceptibility gene (Corder et al., 1993; Lambert et al., 2013). In cognitively healthy participants, presence of the E4 allele has been associated with brain atrophy (Enzinger et al., 2005), and gene dose effects have been reported such that whole brain atrophy rates increased with the number of E4 alleles (Chen et al., 2007). Others, however, have reported a steeper rate of hippocampal atrophy in apoE E4 carriers relative to non-carriers, but no effects on whole brain volume (Moffatt et al., 2000). Still, others have reported little influence of apoeE E4 on change in regional brain volumes (Raz et al., 2010). Thus, despite robust associations between apoE genotype and pathological cognitive aging (e.g., Alzheimer's disease), its role in healthy aging is less clear. Furthermore, it is unlikely that a single genetic factor would account for the observed variability in age-related brain atrophy.

Additional genetic contributions to brain aging include the Human Leukocyte Antigen (HLA) genes. HLA genes, located in the Major Histocompatibility Complex (MHC) of chromosome 6, play a central role in immune system functioning (Meuer et al., 1982). HLA Class II molecules facilitate antigen-specific control of the immune system via production of antibodies and ultimately elimination of pathogens. Successful elimination of pathogens by specific antibody production hinges in part on a match between the pathogen and the HLA protein. In the absence of a match, the pathogen is not eliminated and can persist in the body causing inflammation, cell damage, and autoimmunity (Institute of Medicine, 2012). Indeed, immune-mediated neuroinflammation has been implicated in the development of several age-related diseases including Alzheimer's disease (Heneka et al., 2015; Heppner et al., 2015), and various pathogens have been detected in brain tissues of individuals with Alzheimer's disease (Mawanda and Wallace, 2013), supporting an association between inability to eliminate pathogens and brain disease. Furthermore, recent reports indicate that various loci within the HLA region appear to be involved in Alzheimer's related brain atrophy (Wang et al., 2017). Finally, recent genome-wide association studies have identified several HLA gene variants as promoting susceptibility to Alzheimer's disease including HLA-DRB5/HLA-DRB1 and HLA DRB1*15:01 (Lambert et al., 2013; Steele et al., 2017). Notably, HLA DRB1*15:01 has been associated with several neurological diseases, leading some to suggest it may be associated with pan-neuronal disease susceptibility (Steele et al., 2017).

What, then, about the converse? That is, are there HLA genes that broadly promote protection? In fact, it has been demonstrated that the DRB1 gene is associated with enhanced cognitive abilities among cognitively healthy adults (Payton et al., 2006). HLA genes, however, are highly polymorphic with some DRB1 variants promoting protection and others conferring disease susceptibility. Thus, investigating HLA-disease associations at the protein level provides the most clarity with regard to health outcomes. In the specific case of DRB1, for instance, DRB1*13:01 and DRB1*13:02 which differ only by a single amino acid residue (Hov et al., 2011) have very different disease associations. While some protective effects have been observed for DRB1*13:01 (van der Woude et al., 2010), it has been shown to be a risk factor for various conditions (Fainboim et al., 2001; Hov et al., 2011; Pando et al., 1999). In contrast, DRB1*13:02 appears to exert broadly protective effects, particularly with regard to immune-related disorders (Bettencourt et al., 2015; Furukawa et al., 2017; Hov et al., 2011). Similarly, we have demonstrated protective effects of HLA-DRB1*13:02 on Gulf War Illness (Georgopoulos et al., 2016), a neuroimmune condition (Georgopoulos et al., 2017). Notably, we have demonstrated that the protective effects of DRB1*13:02 extend to brain volume, sparing subcortical atrophy (James et al., 2017) that is characteristic of Gulf War Illness (Christova et al., 2017). Thus, it seems that DRB1*13:02 may confer broad protection against conditions affecting the brain. Given the well-established protective effects of DRB1*13:02, we evaluated in the present study the effect of DRB1*13:02 on brain volume in cognitively healthy women. We hypothesized that DRB1*13:02 carriers would exhibit reduced atrophy relative to non-carriers.

## 2 Materials and Methods

### 2.1 Participants

Seventy-one cognitively healthy women (mean age ± SEM, 54.17 ± 1.23 years, range: 32–69 years) participated in the current study after providing informed consent, in adherence to the Declaration of Helsinki, and were financially compensated for their time. All study protocols were approved by the appropriate Institutional Review Boards. Their cognitive status was assessed using the Montreal Cognitive Assessment (MoCA; http://www.mocatest.org/); all women had MoCA scores > 25, (28.1 ± 0.16, mean ± SEM, N = 71).

### 2.2 HLA Genotyping

DNA isolation was carried out from 3 ml of whole blood drawn in EDTA tubes, using a commercially available kit (ArchivePure cat. 2300730) from 5Prime (distributed by Fisher Scientific or VWR) with an expected yield of 50–150 μg of DNA. The purified DNA samples were sent to Histogenetics (http://www.histogenetics.com/) for high-resolution HLA Sequence-based Typing (SBT; details are given in https://bioinformatics.bethematchclinical.org/HLA-Resources/HLA-Typing/High-Resolution-Typing-Procedures/ and https://bioinformatics.bethematchclinical.org/WorkArea/DownloadAsset.aspx?id=6482). Their sequencing DNA templates are produced by locus- and group-specific amplifications that include exon 2 and 3 for class I (A, B, C) and exon 2 for class II (DRB1, DRB3/4/5, DQB1, and DPB1) and reported as Antigen Recognition Site (ARS) alleles as per ASHI recommendation (Cano et al., 2007).

### 2.3 ApoE Genotyping

DNA samples were genotyped using PCR amplification followed by restriction enzyme digestion (Reymer et al., 1995). Each amplification reaction contained PCR buffer with 15 mmol/L MgCl_2_ ng amounts of genomic DNA, 20 pmol apoE forward (5N TAA GCT TGG CAC GGC TGT CCA AGG A 3N) and reverse (5N ATA AAT ATA AAA TAT AAA TAA CAG AAT TCG CCC CGG CCT GGT ACA C 3N) primers, 1.25 mmol/L of each deoxynucleotide triphosphate, 10% dimethylsulfoxide, and 0.25 μL Amplitaq DNA polymerase. Reaction conditions in a thermocycler included an initial denaturing period of 3 min at 95 C, 1 min at 60 C, and 2 min at 72 C; followed by 32 cycles of 1 min at 95 C, 1 min at 60 C, and 2 min at 72 C; and a final extension of 1 min at 95 C, 1 min at 60 C, and 3 min at 72 C. PCR products were digested with *HhaI* and separated on a 4% Agarose gel which was stained with Ethidium Bromide. Known apoE isoform standards were included in the analysis.

Fifty-three out of 71 participants (74.6%) lacked the apoE4 isoform, whereas 18/71 (25.4%) carried it.

### 2.4 MRI Data Acquisition and Preprocessing

All data were acquired using a 3T MR scanner (Achieva, Philips Healthcare, Best, The Netherlands) with a phased array SENSitivity Encoding (SENSE) 8-channel head coil for reception. For each participant a high resolution T1-weighted image Turbo Field Echo (T1w TFE SENSE) was obtained (168 sagittal slices, TR = 8.1932 ms, TE = 3.752 ms, Acquisition matrix 256 × 256, Flip angle 8°, voxel size 0.9375 × 0.9375 × 1 mm). A T2-weighted image (T2w VISTA HR SENSE) was also obtained (180 slices, TR = 2500 ms, TE = 363.072 ms, acquisition matrix 256 × 256, voxel size = 0.7813 × 0.7813 × 1 mm).

A 704-core High Performance Computing system (CentOS 6.5 Linux, Rocks 6.1.1) with Matlab R2012 (64 bit), Human Connectome Project (HCP humanconnectome.org) pipeline with FreeSurfer (FS; http://surfer.nmr.mgh.harvard.edu) HCP version (freesurfer-hpc) was used for data processing. MRI data with high contrast between gray matter, white matter, and cerebrospinal fluid as well as high spatial resolution are necessary for accurate results. We acquired T1w and T2w images with high spatial resolution (≤1 mm^2^) to achieve precise surface reconstruction. Standard FS software requires only T1w images as input. However, we used a modified version of FS, implemented in the structural HCP pipeline, which utilizes both T1w and T2w images to eliminate uncertainty due to the fact that dura and blood vessels are isointense to gray matter in the T1w image alone. In addition, T2w allows improved pial surface reconstruction (Glasser et al., 2013). Specifically, we used the first 2 structural HCP pipelines, namely PreFreeSurfer and FreeSurfer. One goal of the PreFreesurfer pipeline is to align the T1w and T2w images. PreFreeSurfer pipeline processing was followed by FreeSurfer pipeline processing which is based on FS version 5.2 with improvements. We thus obtained estimated total intracranial volume (eTIV), total gray matter volume, cerebrocortical gray matter volume, and subcortical gray matter volume.

### 2.5 Data Analysis

#### 2.5.1 Basic Analyses

Standard statistical methods were employed to analyze the data using the IBM-SPSS statistical package (version 25), including linear regression. First, the effect of eTIV was removed by regressing the volume against eTIV, and taking the residuals. The effect of age was then estimated in a linear regression where the residuals above were the dependent variable, and age was the independent variable. Such regressions were performed for two groups, namely (1) participants lacking the DRB1*13:02 allele (N = 60, age range 32–69 years), and (2) participants carrying the DRB1*13:02 allele (N = 11, age range 37–68 years). No participant was homozygote for the DRB1*13:02 allele. An estimate of the average percent change in brain volume with age was obtained as the percentage of the regression coefficient for age with respect to the mean volume (adjusted for eTIV).

#### 2.5.2 Assessment of apoE4 Effect

A possible effect of the presence of the apoE4 isoform was assessed by adding an apoE4 binary covariate to the regression model (0 = apoE4 absent, 1 = apoE4 present).

## 3 Results

The age and MoCA scores for each group are given in Table 1. Age and MoCA scores did not differ significantly between groups (ANOVA). All the results below refer to volumes adjusted for eTIV.

**Table 1:** Age and cognitive score of participants (mean ± SEM) for the three DRB1*13 groups.

	DRB1*13:02 absent (N = 60)	DRB1*13:02 present (N = 11)
Age (y)	53.8 ± 1.31	55.36 ± 3.16
MoCA	28.07 ± 0.18	28.18 ± 0.38

### 3.1 Total Gray Matter Volume

#### 3.1.1 No DRB1*13:02

**Fig. 1:**
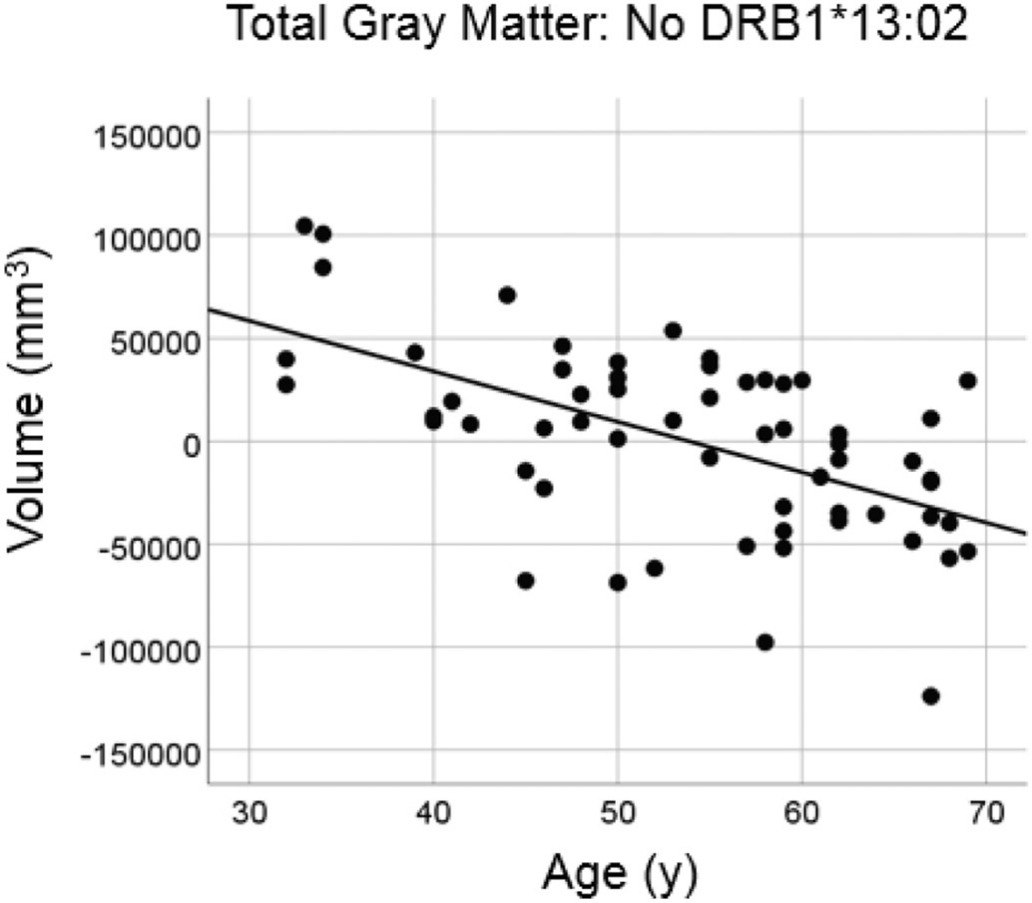
Total brain gray matter volume is plotted against age for the No DRB1*13:02 group (N = 60). Values of volumes are residuals after adjusting for total intracranial volume (eTIV).

There was a highly statistically significant reduction in total gray matter volume with age in the group lacking the DRB1*13:02 allele (Fig. 1; slope = volume reduction rate = −2452.8.7mm^3^/year, P = .000002, R^2^ = 0.325, N = 60), amounting to a reduction rate of −4.2% of the mean volume per decade.

#### 3.1.2 DRB1*13:02

**Fig. 2:**
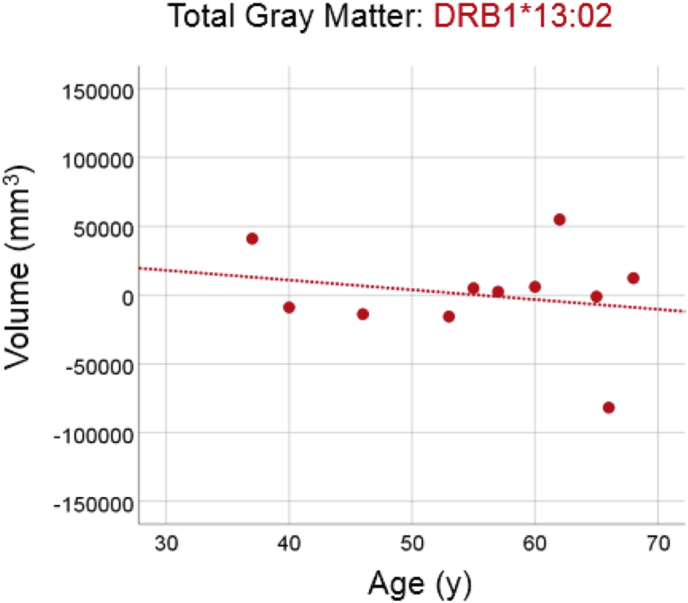
Total brain gray matter volume is plotted against age for the DRB1*13:02 group (N = 11). Conventions are as in Fig. 1. The dotted line indicates that the slope of the fitted line did not differ significantly from zero (see text for details).

There was no statistically significant reduction in total gray matter volume in the group carrying the DRB1*13:02 allele (Fig. 2; slope = −708.6 mm^3^/year, P = 0.529, R^2^ = 0.046, N = 11), amounting to a reduction rate of −1.2% of the mean volume per decade. The slope above was significantly smaller than the one of the No DRB1*13:02 group in the preceding section (P = 0.04).

### 3.2 Total Cortical Gray Matter Volume

#### 3.2.1 No DRB1*13:02

**Fig. 3:**
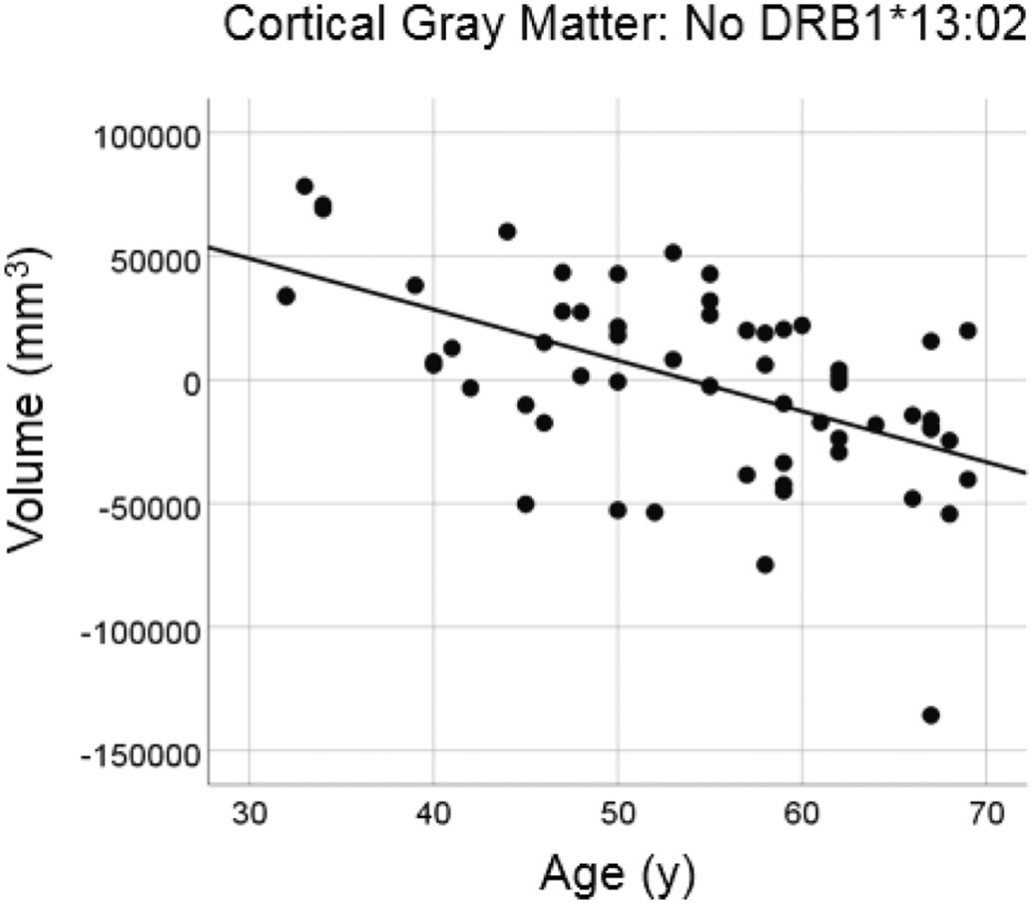
Cortical gray matter volume is plotted against age for the No DRB1*13:02 group. Conventions are as in Fig. 1.

There was a highly statistically significant reduction in total cortical gray matter volume with age in the group lacking the DRB1*13:02 allele (Fig. 3; slope = −2058.8 mm^3^/year, P = 0.00004, R^2^ = 0.310, N = 60), amounting to a reduction rate of −4.7% of the mean volume per decade.

#### 3.2.2 DRB1*13:02

**Fig. 4:**
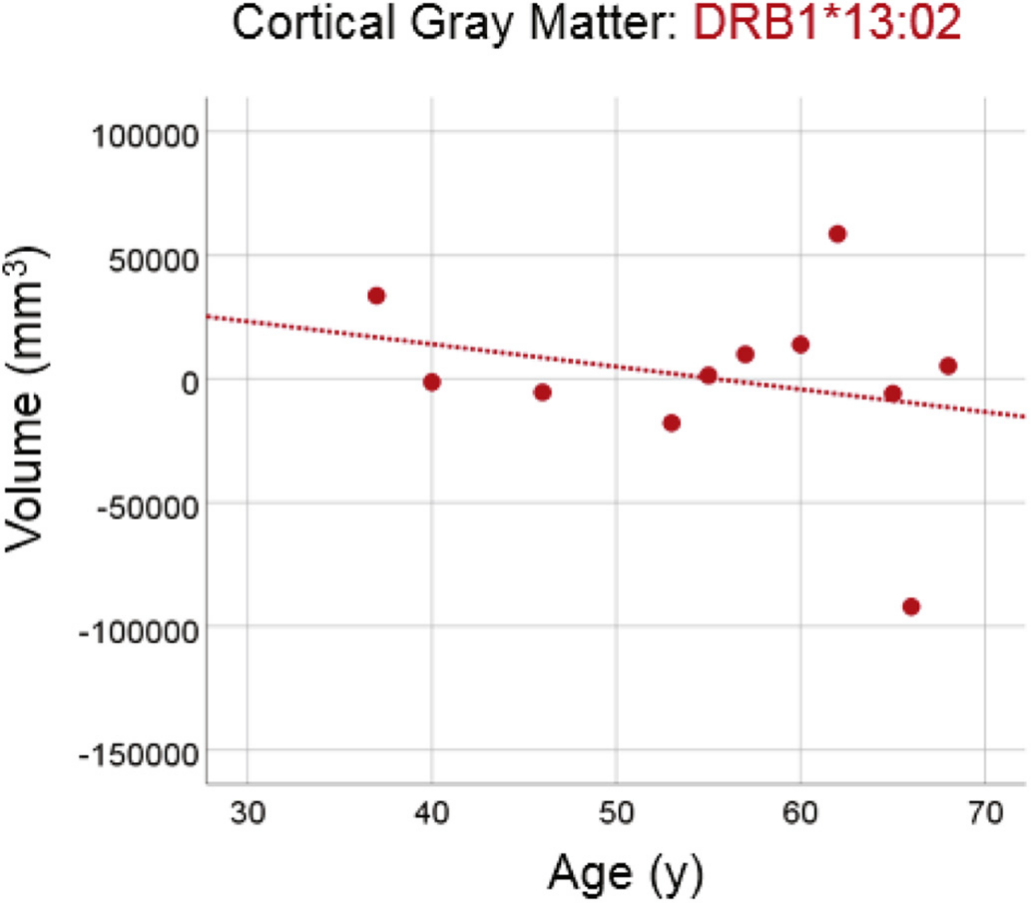
Cortical gray matter volume is plotted against age for the DRB1*13:02 group. Conventions are as in Fig. 2.

There was no statistically significant reduction in total cortical gray matter volume in the group carrying the DRB1*13:02 allele (Fig. 4; slope = −914.3 mm^3^/y, P = 0.443, R^2^ = 0.067, N = 11), amounting to a reduction rate of −2.1% of the mean volume per decade. The slope above was significantly smaller than the one of the No DRB1*13:02 group in the preceding section (P = 0.045).

### 3.3 Total Subcortical Gray Matter Volume

#### 3.3.1 No DRB1*13:02

**Fig. 5:**
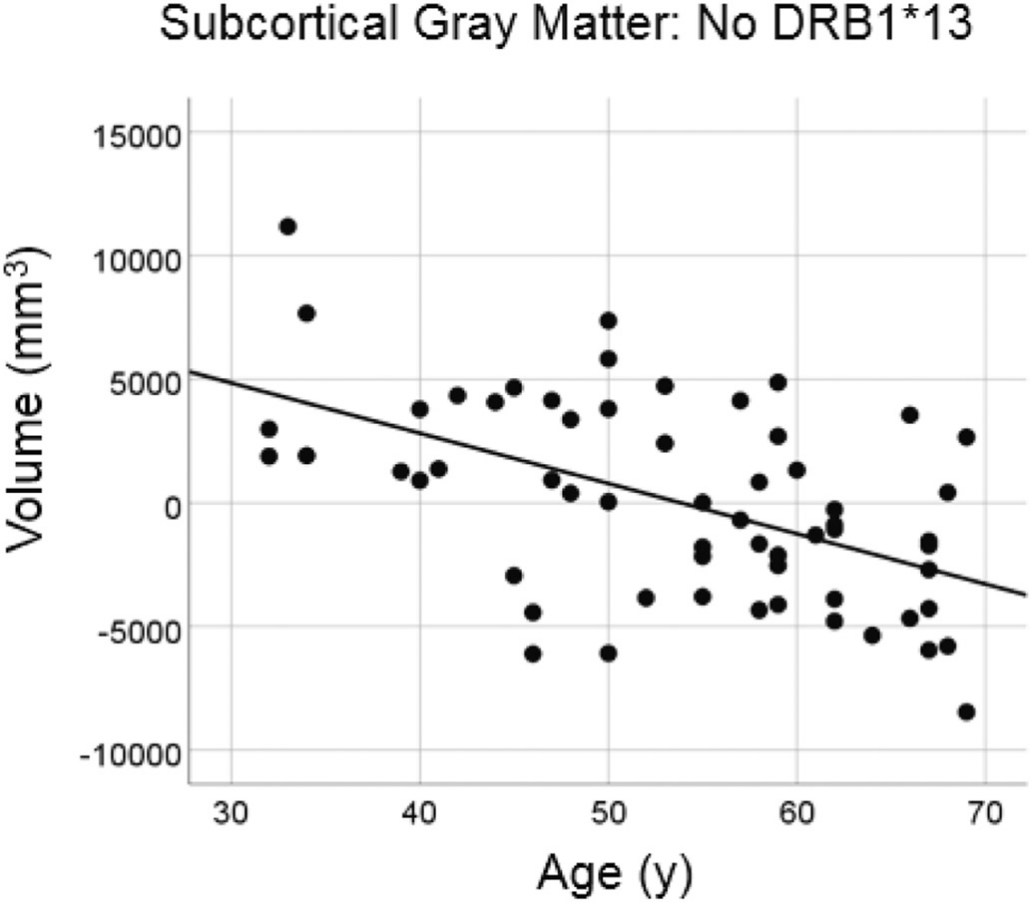
Subcortical gray matter volume is plotted against age for the No DRB1*13:02 group. Conventions are as in Fig. 1.

There was a highly statistically significant reduction in total subcortical gray matter volume with age in the group lacking the DRB1*13:02 allele (Fig. 5; slope = −203.5 mm^3^/year, P = 0.000015, R^2^ = 0.278, N = 60), amounting to a reduction rate of −3.8% of the mean volume per decade.

#### 3.3.2 DRB1*13:02

**Fig. 6:**
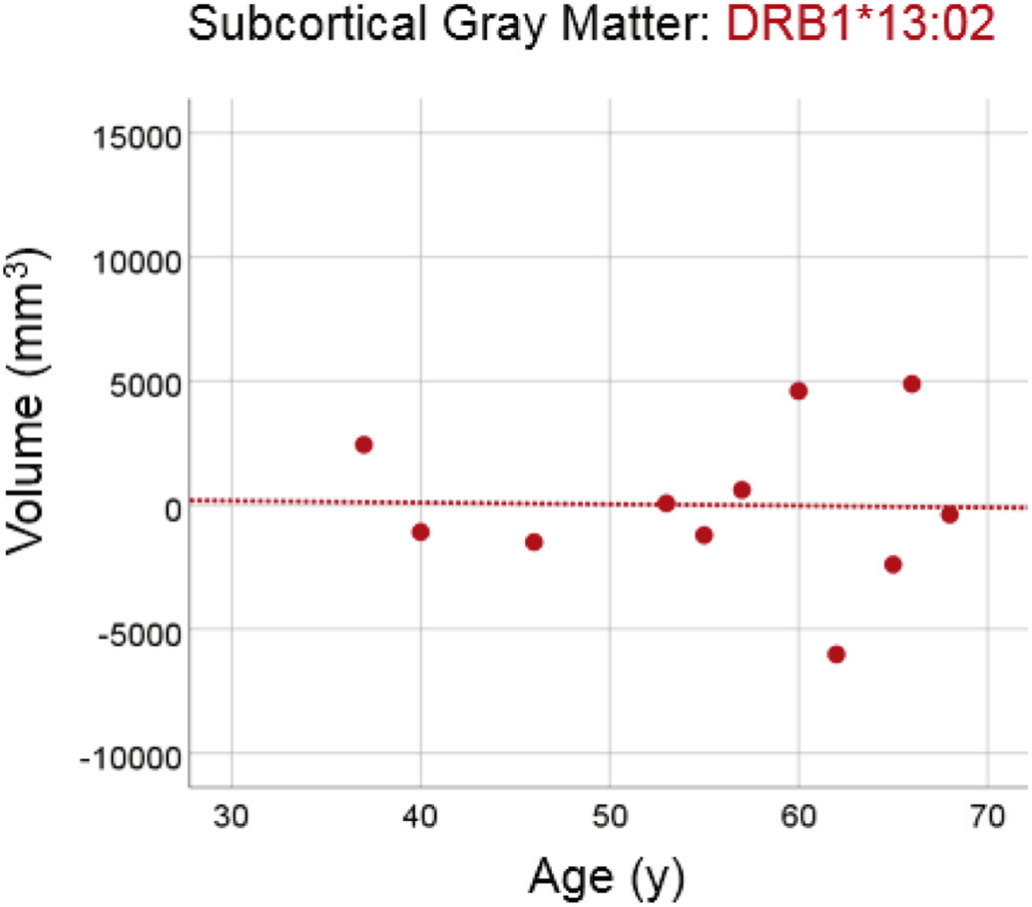
Subcortical brain gray matter volume is plotted against age for the DRB1*13:02 group. Conventions are as in Fig. 2.

There was no statistically significant reduction in total subcortical gray matter volume in the group carrying the DRB1*13:02 allele (Fig. 6; slope = −6.7 mm^3^/year, P = 0.948, R^2^ = 0.001, N = 11), amounting to −0.14% of the mean volume per decade. The slope above was significantly smaller than the one of the No DRB1*13:02 group in the preceding section (P = 0.0005).

### 3.4 Effect of apoE4

The absence or presence of apoE4 isoform was not associated with the frequency of DRB1*13:02 allele (Table 2; *x*_[2]_^2^ = 0.025, P = 0.873). In addition, the apoE4 covariate (added in the regression model together with eTIV and age; see Materials and Methods) was not statistically significant in any analysis.

**Table 2:** Frequency of occurrence of the apoE4 genotype and the two DRB1*13 groups (see text for details.)

		DRB1*13 absent	DRB1*13:02 only	Total
ApoE4	Absent	45	8	53
	Present	15	3	18
	Total	60	11	71

## 4 Discussion

In the present study we investigated the effect of HLA DRB1*13:02 on age-related brain atrophy in cognitively healthy women aged 32–69 years. We found that brains of participants lacking the DRB1*13:02 allele showed a highly statistically significant age-dependent reduction of total gray matter volume; in contrast, participants carrying the DRB1*13:02 allele did not show a significant reduction. Similar effects were observed separately for the volumes of cerebrocortical and subcortical gray matter. These results further extend the protective effect of the DRB1*13:02 on brain gray matter loss in healthy people, as reported in our previous study on Gulf War Illness (James et al., 2017).

The purpose and structure of HLA-DRB1*13:02 provides some insights into the mechanisms that may confer protection against brain atrophy, as observed here. Generally, the goal of HLA Class II molecules (which includes DRB1*13:02) is to eliminate exogenous antigens by leading to the production of antibodies against them. This process involves binding of antigens to a groove formed by polypeptide chains on the HLA molecule in order to create a molecule-peptide complex that is recognized by CD4+ T-lymphocytes thereby stimulating antibody production by plasma cells. Even single amino acid variations alter the peptide-binding specificity and consequently alter disease associations as previously discussed with regard to DRB*13:01 and DRB*13:02 (Hov et al., 2011). With regard to DRB1*13:02, for instance, a simple search of the Immune Epitope Database (Vita et al., 2015) documents several associated antigens in humans, including antigens related to influenza A and hepatitis B and C, while other HLA molecules bind to different antigens. Thus, one's genetic makeup with respect to HLA alleles determines whether there is a match with specific antigens and consequently whether antibodies will be produced and offending antigens eliminated. In the absence of a match, an antigen may persist resulting in potentially deleterious effects including inflammation and possibly autoimmunity (IOM, 2012). This is what we have referred to as the “persistent antigen” hypothesis for Gulf War Illness (James et al., 2017). Although the persistent antigen hypothesis was initially discussed in relation to Gulf War Illness, we suspect it similarly applies to other diseases affecting the brain, including those associated with age-related decline, and likely extends to other organ systems as well.

Thus, we wade into the heated debate regarding whether age-related biological changes are universal or reflective of disease (Bulterijs et al., 2015). While some age-related changes may be universal, our results suggests that brain atrophy may not be. That is, given a certain genetic makeup, age-related brain changes are minimized, as demonstrated in the present study. Similarly, others have reported on “superagers”, namely older adults whose performance on cognitive tests is spared from typical cognitive decline. Recent studies have demonstrated that preserved cognitive functioning among superagers is reflected in preserved cortical integrity. Specifically, it has been demonstrated that superagers (ages 80+) exhibit enhanced cortical thickness and volume compared to age-matched normal agers that is indistinguishable from middle aged (ages 50–65) (Harrison et al., 2012) and even young adults (ages 18–35) (Sun et al., 2016). Perhaps superagers have won the genetic lottery with respect to HLA, permitting successful elimination of common pathogens and, consequently, retention of brain volume and function.

Aging has been referred to as “the consequence of evolutionary neglect, not evolutionary intent” (Olshansky et al., 2002 p. 294). This statement is particularly relevant in light of the highly polymorphic nature of HLA and its purported role in natural selection via adaptive immunity (Meyer et al., 2017; Trowsdale and Knight, 2013). Indeed, HLA gene variations have been associated with various viral diseases (e.g., chicken pox, shingles, cold sores, mononucleosis, mumps) and bacterial infections (tuberculosis, scarlet fever, pneumonia) (Tian et al., 2017) in addition to many autoimmune conditions including diabetes, arthritis, celiac disease, lupus, ankylosing spondylitis, multiple sclerosis, psoriasis, and Crohn's disease (Trowsdale and Knight, 2013). Thus, advantage would be afforded to those with an HLA-profile that maximizes neutralization and eradication of pathogens. We suspect that HLA-DRB1*13:02 may be particularly advantageous and may promote successful aging.

Finally, we would like to entertain an additional hypothesis for a possible neuroprotective role of DRB1*13:02, not mutually exclusive with the persistent antigen hypothesis above. This hypothesis comes from the observation by Davenport et al. (1995) that DRB1*13:02 binds specifically two epitopes from cathepsin S: DPTLDHHWHLWKKTYGKQYKE (21–42) and DPTLDHHWHLWKKTYGKQYK (21–41) (Davenport et al., 1995, Table 1). The binding of the latter peptide to purified HLA-DRB1 was found to be strong for DRB1*13:02 (IC_50,_ μM = 20), weak for DRB1*13:01 (IC_50,_ μM = 100), and practically none for DRB1*01:01 (IC_50,_ μM > 1000) (Davenport et al., 1995, Table 2). Cathepsin S is a protease active in a good range of pH environments, is mainly expressed in the professional antigen-presenting cells, where HLA Class II molecules are also expressed, is involved with antigen presentation to those molecules (Hsing and Rudensky, 2005; Riese et al., 1996), and is upregulated by interferon gamma (IFN-γ) (Beers et al., 2003; van's Gravesande et al., 2002). Cathepsin S is also expressed throughout the brain, and specifically in microglia, where it has been found to increase with aging and in pathological conditions (Wendt et al., 2008). It is also involved in secondary brain damage following traumatic brain injury, where inhibition of cathepsin S had beneficial effects on rescuing brain damage and improving neurobehavioral recovery (Xu et al., 2013).

We hypothesize that the specific binding of DRB1*13:02 to cathepsin S (Davenport et al., 1995) might lead to the production of antibodies that may, ultimately, limit the availability of cathepsin S, thus exerting an indirect neuroprotective effect, especially in conditions where cathepsin S seems to play a detrimental role, as in aging, neuroinflammatory conditions, traumatic brain injury, and other brain diseases, such as Alzheimer's disease (Shi et al., 1994; Munger et al., 1995; Lemere et al., 1995) and amyotrophic lateral sclerosis (Wendt et al., 2008).

## 5 Limitations of the study

The main limitation of this study is the small sample size. Larger samples and longitudinal follow up are needed to further substantiate the neuroprotective role of DRB1*13:02 regarding brain gray matter loss with aging and permit an evaluation of the influence of other genetic, lifestyle, and environmental factors linked to brain atrophy on the protective effect observed here. An additional limitation concerns the fact that the study involved only women participants, and an extension of these findings to men would be important for their generalization. These aspects are currently under investigation in our Brain Resilience Initiative (http://healthybrain.umn.edu/womenshealthybrain.shtml).

## Financial Disclosures

The authors do not report any financial disclosures.

## Author Contributions

Contributed to data collection: PC, SML. Contributed to study design: AG, APG. Contributed to data analysis: PC, APG, LMJ, BEE, AG. Wrote the paper: LMJ, APG, AG. Contributed to editing the paper: All.

## Role of the Funding Source

Partial funding for this study was provided by the University of Minnesota (the Kunin Professorship for Women's Healthy Brain Aging, the Brain and Genomics Fund, the McKnight Presidential Chair of Cognitive Neuroscience, and the American Legion Brain Sciences Chair). The sponsors had no role in the current study design, analysis or interpretation, or in the writing of this paper. The contents do not represent the views of the U.S. Department of Veterans Affairs or the United States Government.

## Funding

U.S. Department of Veterans Affairs, and University of Minnesota.
